# Multimodality approach of perioperative ^18^F-FDG PET/CT imaging, intraoperative ^18^F-FDG handheld gamma probe detection, and intraoperative ultrasound for tumor localization and verification of resection of all sites of hypermetabolic activity in a case of occult recurrent metastatic melanoma

**DOI:** 10.1186/1477-7819-6-1

**Published:** 2008-01-10

**Authors:** Stephen P Povoski, Nathan C Hall, Edward W Martin, Michael J Walker

**Affiliations:** 1 Division of Surgical Oncology, Department of Surgery, Arthur G. James Cancer Hospital and Richard J. Solove Research Institute and Comprehensive Cancer Center, The Ohio State University, Columbus, OH 43210, USA; 2 Section of PET, Division of Nuclear Medicine, Department of Radiology, The Ohio State University, Columbus, OH, 43210, USA

## Abstract

**Background:**

The use of diagnostic ^18^F-fluorodeoxyglucose (^18^F-FDG) positron emission tomography/computed tomography (PET/CT) imaging for the staging, restaging, and treatment monitoring of melanoma patients has become a well-recognized standard of care. It plays a key role in detecting sites of occult disease and is widely utilized in the medical and surgical planning of such patients. In the current report, we describe an innovative multimodality approach of perioperative ^18^F-FDG PET/CT imaging, intraoperative ^18^F-FDG handheld gamma probe detection, and intraoperative ultrasound for tumor localization and verification of resection of all sites of hypermetabolic tumor foci in a case of occult recurrent metastatic melanoma.

**Case presentation:**

This report discusses a case of occult recurrent metastatic melanoma, isolated to three separate sites within the subcutaneous tissues of the left thigh region, which was not clinically apparent but was found on diagnostic restaging whole body ^18^F-FDG PET/CT scan utilizing an intravenous injection of 14.8 mCi ^18^F-FDG. Then, on the day of surgery, the patient received an intravenous injection of 12.8 mCi ^18^F-FDG. A multimodality approach of intraoperative handheld gamma probe detection, intraoperative ultrasound tumor localization, specimen PET/CT imaging, and postoperative PET/CT imaging was utilized for accomplishing and verifying the excision of all three sites of occult recurrent metastatic melanoma within the left thigh region.

**Conclusion:**

This innovative multimodality approach of perioperative ^18^F-FDG PET/CT imaging, intraoperative ^18^F-FDG handheld gamma probe detection, and intraoperative ultrasound is promising combined technology for aiding in tumor localization and verification of excision and may ultimately impact positively upon long-term outcome of selected patients.

## Background

In the year 2007, within the United State alone, it is estimated that approximately 60,000 cases of melanoma will be diagnosed and approximately 8,100 people will die of this disease [1]. Early detection and appropriate surgical intervention with wide excision of the primary lesion and evaluation of suspect lymph node basins remain the hallmarks of the initial management strategy for melanoma [2]. Still, the risk of developing both locoregional recurrence and distant recurrence remains a legitimate concern and consequently portends a poor prognostic outcome [3]. Despite advances in systemic therapies for metastatic melanoma, surgical resection of limited recognizable recurrent disease is considered appropriate and is often the preferred management strategy [2].

Diagnostic ^18^F-fluorodeoxyglucose (^18^F-FDG) positron emission tomography/computed tomography (PET/CT) imaging has become a well-established method for staging, restaging, and monitoring response to therapy of melanoma patients, and is widely accepted as a standard of care [2,4,5]. In this regard, this technology plays a key role in detecting sites of occult disease and is widely utilized in the medical and surgical planning of such patients [2,4-6].

The current application of ^18^F-FDG PET/CT imaging for melanoma patients is generally that of diagnostic image acquisition at the time of the original evaluation in those individuals considered at an elevated risk for regional and/or distant disease. In those instances when regional and/or distant disease can potentially be surgically approached, this current practice of diagnostic image acquisition at the time of the original patient evaluation provides only a static roadmap for guiding the surgical approach, but does not provide the surgeon with real-time intraoperative information on tumor location and verification of tumor resection. Recently, the application of intraoperative gamma probe detection in melanoma patients after preoperative injection of an intravenous dose of ^18^F-FDG has been reported by several groups of investigators [7-11]. Furthermore, the specific application of a combined approach of preoperative ^18^F-FDG PET/CT imaging and intraoperative gamma probe detection for a case of recurrent melanoma has been recently reported by Carrera et al [11]. Applying this approach to recurrent melanoma, as well as further development and refinement of such innovative approaches for perioperatively detecting and intraoperatively directing the surgeon in identifying and removing all sites of disease may ultimately translate into improved long-term outcome of selected patients.

In the current Technical Innovations report, we describe an innovative multimodality approach of perioperative ^18^F-FDG PET/CT imaging, intraoperative ^18^F-FDG handheld gamma probe detection, and intraoperative ultrasound for tumor localization and verification of resection of all sites of hypermetabolic activity in a case of occult recurrent metastatic melanoma.

## Case presentation

The case presented is that of a 50 year-old Caucasian female with isolated recurrence of metastatic melanoma to the subcutaneous tissues of her left thigh. Ten years prior to her current presentation, she underwent a wide excision and skin grafting of her left distal thigh region and a superficial left groin lymph node dissection for a 2.3 mm malignant cutaneous melanoma with 22 negative lymph nodes. She received no adjuvant therapy and subsequently continued routine follow-up by her surgeon.

Twenty-five months prior to her current presentation, she developed two skin nodules located approximately 4 cm distal to the previous skin graft on her distal thigh region. A wide excision of her left distal thigh region was subsequently performed. A diagnostic whole body ^18^F-FDG PET scan was performed, utilizing an intravenous injection of 13.9 mCi ^18^F-FDG, that revealed a solitary hypermetabolic focus within the anteriomedial left mid thigh region (peak SUV of 40.1) which was not palpable on clinical examination. No other hypermetabolic foci were identified elsewhere in her body. As a result, the patient subsequently (23 months prior to her current presentation) underwent isolated left lower extremity hyperthermic limb perfusion with melphalan and a concomitant left deep groin lymph node dissection.

A six-month follow-up (17 months prior to her current presentation) diagnostic whole body ^18^F-FDG PET/CT scan was performed, utilizing an intravenous injection of 16.3 mCi ^18^F-FDG, and redemonstrated a solitary hypermetabolic focus within the subcutaneous tissues of the anteriomedial left mid thigh region, with a peak SUV of 7.6. No other hypermetabolic foci were identified elsewhere in her body. Subsequently (15 months prior to her current presentation), CT guided wire localization and wide excision of the nonpalpable subcutaneous focus of disease in her anteriomedial left mid thigh region was performed.

**Figure 1:**
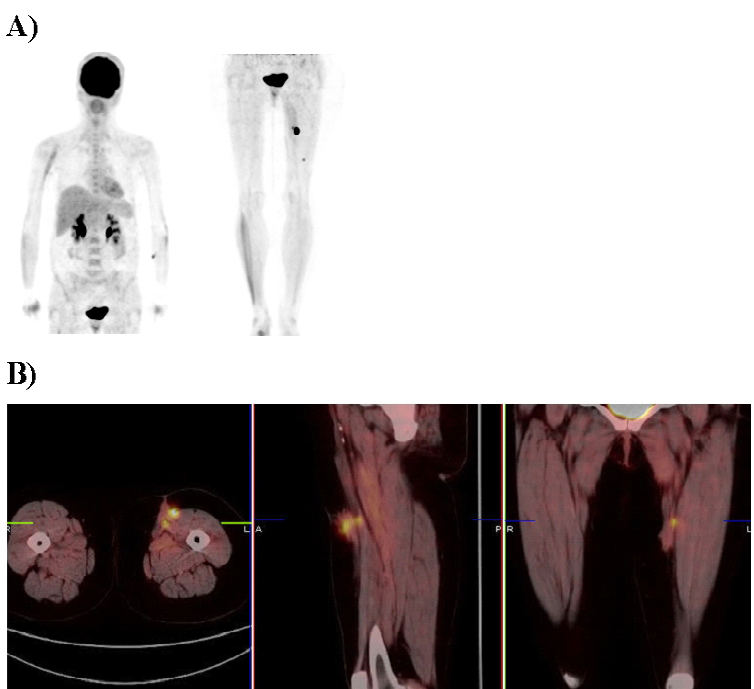
Preoperative PET maximum intensity projection (A) and preoperative cross sectional fused PET/CT images (B) of a patient with recurrent metastatic melanoma to the left thigh. The preoperative PET/CT scan revealed three hypermetabolic foci within the left thigh.

The patient continued routine follow-up by her surgeon. One month prior to her current presentation, the patient underwent a routine follow-up diagnostic restaging whole body ^18^F-FDG PET/CT scan (Figure 1) on a Siemens Biograph 16 PET/CT unit (Knoxville, TN, USA) utilizing an intravenous injection of 14.8 mCi ^18^F-FDG. The scan demonstrated three foci of hypermetabolic activity within the subcutaneous tissues of the anterior left thigh region. Two closely approximated hypermetabolic foci (peak SUV of 25.3) were located in the subcutaneous tissues of the anteriomedial left mid thigh region and one hypermetabolic focus (peak SUV of 3.4) was located in the subcutaneous tissues of the anterior lower one-third of the left thigh region. No other hypermetabolic foci were identified elsewhere in her body. On clinical exam, no visible or palpable abnormalities were noted in the left thigh region or elsewhere.

On the day of surgery, a dose of 12.8 mCi ^18^F-FDG was injected intravenously into a peripheral vein at approximately 80 minutes prior to the start time of the surgical procedure, as per our ^18^F-FDG and PET/CT protocols previously described [12,13]. The patient was subsequently taken to the operating room. Intraoperatively, a handheld gamma probe (Neoprobe neo2000 unit, Neoprobe Corporation, Dublin, Ohio, USA) was used to attempt localization of the three areas of increased ^18^F-FDG uptake within the left thigh region. Initially, one predominant site of ^18^F-FDG activity was transcutaneously identified with the gamma probe within the anteriomedial left mid thigh region. Surgical excision of the subcutaneous tissue (measuring 16.0 × 7.0 × 3.0 cm in size) of this area was undertaken. Post-excision evaluation of the excision bed within this region was performed with the gamma probe and revealed residual increased ^18^F-FDG activity above background. Therefore, gamma probe directed excision of additional deeper subcutaneous tissue (measuring 3.5 × 2.0 × 1.0 cm in size) of this area was undertaken. Further post-excision reevaluation of the excision bed within this region was again performed with the gamma probe and revealed no ^18^F-FDG activity above background.

**Figure 2:**
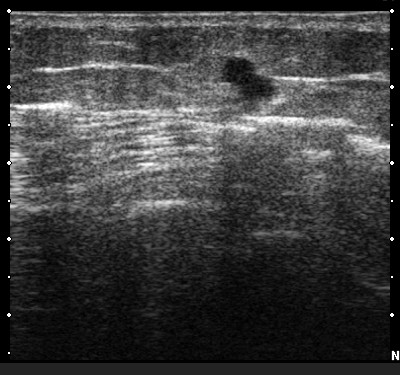
Intraoperative ultrasound showing an 8 mm hypoechoic lesion identified within the subcutaneous tissues of an area of generally increase ^18^F-FDG activity within the anterior lower one-third of the left thigh region.

Attention was then directed to the anterior lower one-third of the left thigh region where the third hypermetabolic focus was located in the subcutaneous tissues based on the previous diagnostic whole body ^18^F-FDG PET/CT scan. With the gamma probe, difficulty was encountered transcutaneously distinguishing a finite site of ^18^F-FDG activity that was distinct from that of the underlying background muscular and vascular blood pool ^18^F-FDG activity. Therefore, intraoperative ultrasound was performed using a Hitachi HI VISION™ 6500 ultrasound system (Hitachi Medical Systems America, Inc., Twinsburg, Ohio, USA) with a variable frequency linear transducer EUP-L54M (range 10.0 to 13.0 MHz) (Hitachi Medical Systems America, Inc., Twinsburg, Ohio, USA). An 8 mm hypoechoic ultrasound lesion (Figure 2) was identified within the subcutaneous tissues coinciding with the area of generally increase ^18^F-FDG activity within the anterior lower one-third of the left thigh region seen on the previous diagnostic whole body ^18^F-FDG PET/CT scan. More localized increase ^18^F-FDG activity was verified within this same region with the gamma probe and surgical excision of subcutaneous tissue (measuring 4.2 × 4.0 × 1.0 cm in size) of this area was undertaken. Post-excision evaluation of the excision bed within this region was performed with the gamma probe and revealed no ^18^F-FDG activity above background. Ex vivo gamma probe evaluation revealed increased ^18^F-FDG activity within the excised subcutaneous tissue. Likewise, ex vivo ultrasound evaluation revealed the corresponding hypoechoic ultrasound lesion within the excised subcutaneous tissue.

**Figure 3:**
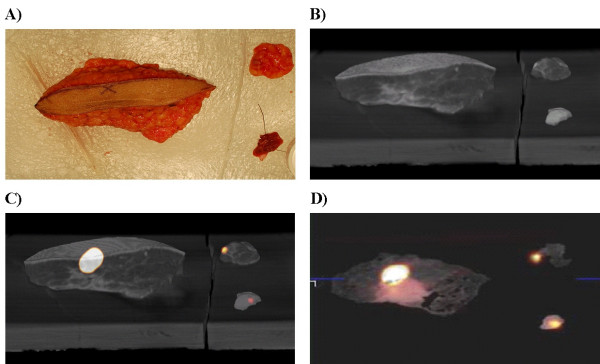
Digital photograph of the three surgical specimens resected from the left thigh (A). Three dimensional CT reconstruction alone of the three surgical specimens from the left thigh (B). Three dimensional CT reconstruction of the three surgical specimens from the left thigh fused with PET activity (C). Cross sectional specimen fused PET/CT images of the melanoma metastases in the left thigh revealing three hypermetabolic foci (D).

All three resected specimens were then transported to the nuclear medicine department and imaged with the clinical PET/CT scanner (Figure 3) at a time of approximately 210 minutes after the original ^18^F-FDG injection. Specimen PET/CT imaging revealed the presence of three hypermetabolic foci, corresponding to the three sites of excised subcutaneous tissue that represented the three hypermetabolic areas in the subcutaneous tissues of the left thigh region that were originally visualized on the preoperative diagnostic whole body ^18^F-FDG PET/CT scan. The specimens were then transported back to the operating room in order to be sent to and processed by surgical pathology for standard pathologic evaluation.

**Figure 4:**
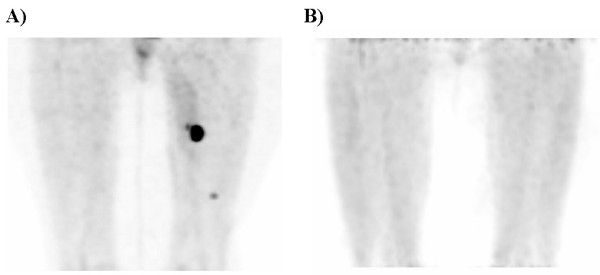
Preoperative PET maximum intensity projection in the anterior projection view (A) and postoperative PET maximum intensity projection in the anterior projection view (B). The postoperative PET/CT scan revealed verification of resection of hypermetabolic foci previously noted on the preoperative scan.

Postoperatively, the patient was recovered uneventfully in the post-anesthesia care unit. After postoperative standard stabilization and recovery (at a time of approximately 120 minutes after the completion of the surgical procedure and at a time of approximately 340 minutes after the original ^18^F-FDG injection), she was subsequently taken to the nuclear medicine department and re-imaged with PET/CT scan without administration of an additional dose of ^18^F-FDG. The postoperative PET/CT scan demonstrated no residual sites of hypermetabolic activity, verifying excision of the all three previously visible sites of hypermetabolic activity within the left thigh region (Figure 4).

Pathologic evaluation of the resected specimens revealed three separate sites of malignant melanoma. This included a 18 mm nodule of malignant melanoma that corresponded to the first excised focus, representing the larger of the two areas in the subcutaneous tissues of the anteriomedial left mid thigh region which demonstrated a peak SUV of 25.3 on the preoperative diagnostic whole body ^18^F-FDG PET/CT scan. Likewise, an additional 8 mm nodule of malignant melanoma was identified that corresponded to the second excised focus, representing the smaller of the two area in the subcutaneous tissues of the anteriomedial left mid thigh region which demonstrated a peak SUV of 25.3 on the preoperative diagnostic whole body ^18^F-FDG PET/CT scan. Finally, a 6 mm nodule of malignant melanoma was identified that corresponded to the third excised focus, representing the area in the subcutaneous tissues of the anterior lower one-third of the left thigh region which demonstrated a peak SUV of 3.4 on the preoperative diagnostic whole body ^18^F-FDG PET/CT scan.

At the time of the publication of this Technical Innovations report, the patient is currently six months out from the above-described innovative multimodality approach for tumor localization and verification of resection of all sites of hypermetabolic activity and appears to be without any evidence of further disease.

## Discussion

In the current Technical Innovations report, we describe a case of occult recurrent metastatic melanoma that nicely illustrates an innovative multimodality approach of perioperative ^18^F-FDG PET/CT imaging, intraoperative ^18^F-FDG handheld gamma probe detection, and intraoperative ultrasound for tumor localization and verification of resection of all sites of hypermetabolic activity. It is our contention [12-14], as well as others [7-11,15-17], that the application of ^18^F-FDG-directed technology should be utilized far-beyond its initial intention of diagnostic imaging of patients at the time of the original evaluation and should be applied to the operative arena for guiding the surgical approach by providing the surgeon with real-time intraoperative information on tumor location and verification of tumor resection.

Several critical points with regards to this innovative multimodality approach were brought to light in this particular case. First, the initial ability of diagnostic whole body ^18^F-FDG PET/CT imaging to identify, when conventional techniques failed, the three site of hypermetabolic activity was instrumental in allowing successful management of a case of occult recurrent metastatic melanoma. Second, intraoperative ^18^F-FDG gamma probe detection was able to initially demonstrate the larger predominant hypermetabolic site of disease within the anteriomedial left mid thigh region, After excision of this first predominant hypermetabolic focus, the gamma probe was able to identify that the second smaller hypermetabolic focus was still present, thus ultimately guiding successful excision of this second smaller hypermetabolic focus. Third, intraoperative ultrasound was critical in identifying the third site of disease located within the anterior lower one-third of the left thigh region which was less metabolically active and which was not initially easily distinguishable from the muscle and blood pools by intraoperative ^18^F-FDG gamma probe detection. Fourth, the re-application of the gamma probe after excision of the presumed third site of disease located within the anterior lower one-third of the left thigh region was critical to verifying that the ultrasound-detected lesion excised was, in fact, the third hypermetabolic focus of disease. Fifth, specimen PET/CT was critical for verifying that each of the three surgically resected tissue specimens contained the corresponding sites of hypermetabolic activity seen on the original diagnostic whole body ^18^F-FDG PET/CT scan. Sixth, the immediate postoperative PET/CT scan clearly demonstrated no residual sites of hypermetabolic activity within the corresponding excision beds, thus verifying excision of the all three previously visible sites of hypermetabolic activity within the left thigh region

## Conclusion

The innovative multimodality approach of perioperative ^18^F-FDG PET/CT imaging, intraoperative ^18^F-FDG handheld gamma probe detection, and intraoperative ultrasound that is described in the current Technical Innovations report is a promising combined technology for aiding in tumor localization and verification of excision and may ultimately impact positively upon long-term outcome of selected patients. Each component of this innovative multimodality approach is technically feasible and should be readily available to all practicing surgeons that have ^18^F-FDG capabilities at their medical facilities. We strongly believe that the success of this innovative multimodality approach will require the future availability of some sort of portable PET/CT scanning device within the operating room arena that would aid significantly in the immediate, real-time intraoperative verification of complete removal of all hypermetabolic activity within the surgical resection field and would allow the surgeon to make further intraoperative decisions about the need for additional surgical resection.

## Abbreviations

^18^F-FDG, ^18^F-fluorodeoxyglucose; 

PET/CT, positron emission tomography/computed tomography; 

SUV, standardized uptake value; 

MRI, magnetic resonance imaging.

## Competing interests

The author(s) declare that they have no competing interests.

## Authors' contributions

SPP organized, wrote, and revised the manuscript. NCH was the nuclear medicine physician who prepared the images for this report. EWM was the supervising senior surgeon for the entire project. MJW was the surgeon on this case. NCH, EWM, and MJW assisted in the writing and editing of this manuscript. All of the authors have approved the final version of this manuscript.
